# Cataracts and Microphthalmia Caused by a Gja8 Mutation in Extracellular Loop 2

**DOI:** 10.1371/journal.pone.0052894

**Published:** 2012-12-26

**Authors:** Chun-hong Xia, Bo Chang, Adam M. DeRosa, Catherine Cheng, Thomas W. White, Xiaohua Gong

**Affiliations:** 1 School of Optometry and Vision Science Program, University of California, Berkeley, California, United States of America; 2 The Jackson Laboratory, Bar Harbor, Maine, United States of America; 3 Physiology and Biophysics, State University of New York – Stony Brook, Stony Brook, New York, United States of America; University of Missouri-Columbia, United States of America

## Abstract

The mouse semi-dominant *Nm2249* mutation displays variable cataracts in heterozygous mice and smaller lenses with severe cataracts in homozygous mice. This mutation is caused by a *Gja8^R205G^* point mutation in the second extracellular loop of the Cx50 (or α8 connexin) protein. Immunohistological data reveal that Cx50-R205G mutant proteins and endogenous wild-type Cx46 (or α3 connexin) proteins form diffuse tiny spots rather than typical punctate signals of normal gap junctions in the lens. The level of phosphorylated Cx46 proteins is decreased in *Gja8^R205G/R205G^* mutant lenses. Genetic analysis reveals that the Cx50-R205G mutation needs the presence of wild-type Cx46 to disrupt lens peripheral fibers and epithelial cells. Electrophysiological data in *Xenopus* oocytes reveal that Cx50-R205G mutant proteins block channel function of gap junctions composed of wild-type Cx50, but only affect the gating of wild-type Cx46 channels. Both genetic and electrophysiological results suggest that Cx50-R205G mutant proteins alone are unable to form functional channels. These findings imply that the *Gja8^R205G^* mutation differentially impairs the functions of Cx50 and Cx46 to cause cataracts, small lenses and microphthalmia. The *Gja8^R205G^* mutation occurs at the same conserved residue as the human *GJA8^R198W^* mutation. This work provides molecular insights to understand the cataract and microphthalmia/microcornea phenotype caused by *Gja8* mutations in mice and humans.

## Introduction

Cataracts, defined as any opacity in the eye lens, remain the leading cause of blindness worldwide. Genetic studies of gene mutations are important for understanding the molecular bases of cataract formation [1], [2], [3]. The lens is comprised of a bulk of elongated fiber cells covered by a monolayer of epithelial cells on the anterior hemisphere. Intercellular gap junction channels connect lens fiber cells and epithelial cells, and provide vital pathways for the transport of important metabolites, ions and fluid needed for lens growth and transparency [4], [5]. Gap junction channels are composed of transmembrane protein subunits known as connexins [6]. Each connexin subunit can be divided into four transmembrane domains, three intracellular domains (amino terminal, carboxy terminal and cytoplasmic loop) and two extracellular loops [7]. Six connexin proteins oligomerize to form a connexon (or hemichannel) [8]. Connexons can be of uniform (homomeric) or varying (heteromeric) connexin composition. Gap junctions are formed when the extracellular domains of two heteromeric or homomeric connexons from adjacent cells dock, creating an intercellular passage for the diffusion of small molecules between the cytoplasm of neighboring cells [9]. Gap junctions can be homotypic channels (two identical connexons consisting of one type of connexin subunits), heteromeric channels (connexons consisting of different types of connexin subunits) or heterotypic channels (connexons each containing a different connexin subunit) [6]. Altering connexin subunit composition affects both the permeability and electrophysiological properties of gap junctions. Members of connexin gene family are utilized in almost all organs and cell types [10]. Mutations of connexin gene family members cause various types of diseases in the cardiovascular system, nervous system, skin and eyes in animals and humans [11], [12], [13], [14].

Lens gap junction channels can be formed by at least three types of connexin subunits encoded by three different genes, Cx43 or α1 connexin encoded by the *Gja1* gene [15], Cx46 or α3 connexin by the *Gja3* gene and Cx50 or α8 connexin by the *Gja8* gene. These connexins have distinct and redundant expression in the lens [16], [17]. In this manuscript, we have selected standard genetic nomenclature *Gja8* and *Gja3* for describing genes, and will use Cx50 and Cx46 for proteins. The Cx43 protein is predominantly expressed in lens epithelial cells. The Cx46 protein is mainly expressed in lens fiber cells, while Cx50 is expressed in both epithelial and fiber cells. In addition, the Cx23 protein, encoded by *Gje1* or *Gjf1*, is only expressed in lens primary fiber cells. A *Gjf1* mutation affects early lens development and causes a variable small-eye phenotype in mice [18]. However, it is unclear whether Cx23 can form gap junction channels [19].

Molecular and cellular mechanisms for the function and regulation of gap junction communication in lens growth and transparency are still far from fully understood. It has been hypothesized that the gap junction network maintains lens homeostasis by providing the outflow pathway in a lens circulation model [4]. Thus, a disruption of these intercellular pathways leads to physiological and/or growth anomalies, such as cataracts and smaller lenses [20]. The deletion of *Gja3* results in recessive nuclear cataracts in mice [21], while a loss of *Gja8* causes recessive phenotypes of small lenses and mild nuclear opacities [16], [22]. Knock-in mice with the genetic replacement of *Gja8* with *Gja3*, which express wild-type *Gja3* from the *Gja8* promoter, have clear lenses but cannot rescue the reduction of lens size caused by the absence of *Gja8* [17]. Further studies have demonstrated that the knock-in *Gja3* alone is sufficient to maintain lens transparency [23].

Almost all point mutations in *Gja3* and *Gja8* lead to variable dominant cataracts in mice and humans [4]. Studies of these point mutations suggest that mutant connexin proteins not only have impaired function, but may also act as dominant negative inhibitors to affect channel properties of other wild-type connexin subunits and may trigger a stress response to disrupt cellular functions [24], [25], [26], [27]. Thus, the investigation of new point mutations in these connexin genes is important for revealing new information about the assembly, regulation and function of gap junction channels as well as for elucidating novel cellular responses to the presence of mutant connexin proteins *in vivo*.

In this study, we have identified a dominant cataract mouse mutation with microphthalmia, caused by the *Gja8^R205G^* point mutation. This mutant mouse line was named as new mutant number 2249 (*Nm2249*) after its cataract phenotype was confirmed in The Jackson Laboratory [28]. This is the first point mutation identified in extracellular loop 2 of the Cx50 protein in mice. We have investigated the molecular and cellular mechanisms for why and how this new mutation causes similar and distinct ocular phenotypes in comparison with previously reported *Gja8* knockout and other *Gja8* point mutations in the N-terminus and extracellular loop 1 *in vivo*. We have also examined electrophysiological properties of gap junction channels formed by homomeric, heteromeric, homotypic and heterotypic interactions of mutant connexin protein subunits with or without wild-type connexin protein subunits *in vitro*. Our results provide some insights for understanding the cataract-microcornea syndrome caused by a similar *Gja8* mutation in humans [29].

## Results

### Identification of a novel *Gja8* mutation that causes semi-dominant cataracts

**Figure 1:**
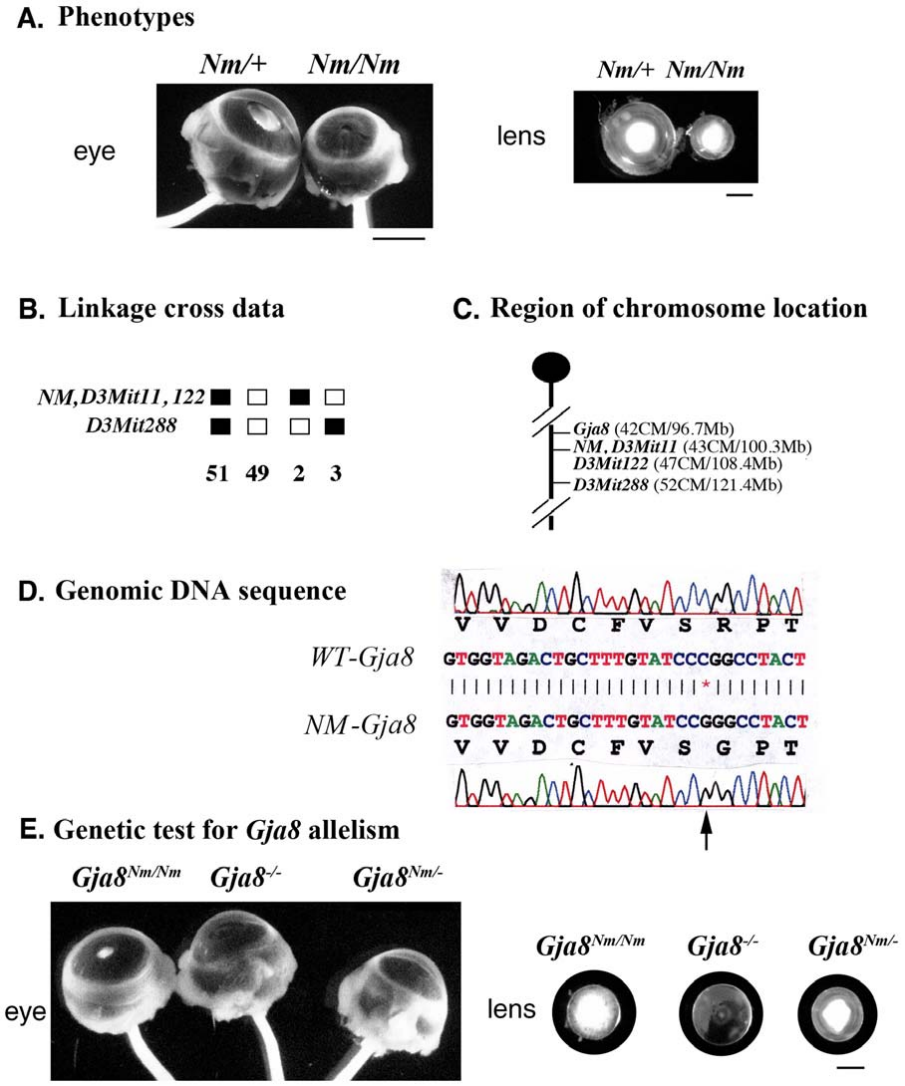
Identification of the *Gja8^R205G^* mutation in *Nm2249* mice. (**A**) Photos of eyes and lenses of *Nm2249* heterozygous (*Nm/+*) and *Nm2249* homozygous (*Nm/Nm*) mutant mice in the CWXS/Agl strain background at the age of 3 weeks. Scale bars, 1 mm. (**B**) Genome-wide screening data of the phenotypes and genotypes of 105 backcrossed mice, generated between mutant *Nm2249* and wild-type CAST/Ei mice. The *Nm2249* mutation was mapped to a region near D3Mit11 and D3Mit122 on chromosome 3. (**C**) The *Gja8* gene is near the region where the *Nm2249* mutation was mapped. (**D**) DNA sequencing data confirmed a missense mutation (C to G) in the *Gja8* gene of *Nm2249* mutant (*Nm-Gja8*), which resulted in the arginine at codon 205 of wild-type *Gja8* (*WT-Gja8*) being replaced by a glycine (R205G). (**E**) Homozygous *Gja8^Nm2249/Nm2249^* mutant mice were bred with the *Gja8^−/−^* knockout mice for a *Gja8* allelic test. Phenotypic comparison of eyes and lenses of *Gja8^Nm2249/Nm2249^, Gja8^Nm2249/−^* and *Gja8^−/−^* mice at the age of 3 weeks was shown. The similarity of small lens size in these mutant mice further indicated that *Gja8^R205G^* is the causative mutation in *Nm2249* mice. Scale bars, 1 mm.

An autosomal semi-dominant cataractous mouse line, *Nm2249*, was first observed in a CWXS/Agl inbred strain. Both heterozygous and homozygous mutant mice had dense nuclear cataracts while only homozygous mutant mice developed microphthalmia (Figure 1A). A genome-wide linkage analysis was used to map this mutation into a region on mouse chromosome 3, where the *Gja8* gene is located (Figure 1B and 1C). DNA sequencing data confirmed that a missense mutation (C to G) at codon 205 resulted in the arginine residue being replaced by a glycine (R205G) in the Cx50 protein (Figure 1D). An allelic test with *Gja8* knockout (−/−) mice revealed that, in contrast to normal eye size of Nm2249/+ heterozygous mutant mice (Figure 1A), mutant mice (*Nm2249/*−) with one *Nm2249* allele and one *Gja8* knockout allele had phenotypes similar to *Nm2249/Nm2249* homozygous mutant mice (Figure 1E), including microphthalmia and small lenses with dense nuclear cataracts. Thus, the *Nm2249* mutation was named *Gja8^R205G^*.

**Figure 2:**
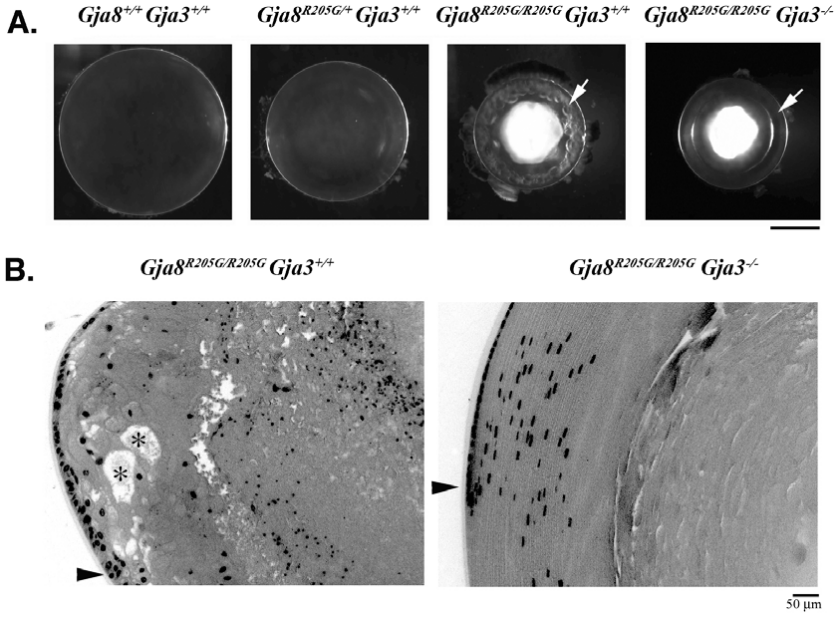
Endogenous Cx46 influences cataract formation caused by the *Gja8^R205G^* mutation. (**A**) Lens photos of wild-type (*Gja8^+/+^ Gja3^+/+^*), *Gja8^R205G^* heterozygous (*Gja8^R205G/+^Gja3^+/+^*), *Gja8^R205G^* homozygous (*Gja8^R205G/R205G^ Gja3^+/+^*), and *Gja8^R205G^ Gja3* double mutant (*Gja8^R205G/R205G^ Gja3^−/−^*) mice at the age of 3 weeks. These mutant lines were bred into the C57BL/6J strain background. While the *Gja8^R205G^* homozygous (*Gja8^R205G/R205G^ Gja3^+/+^*) lens revealed vacuole-like defects in the lens periphery (indicated by a white arrow), the *Gja8^R205G/R205G^ Gja3^−/−^* double mutant lens showed a clear lens periphery (indicated by a white arrow). Scale bar, 1 mm. (**B**) Histological sections of *Gja8^R205G^* homozygous mutant (*Gja8^R205G/R205G^ Gja3^+/+^*) and *Gja8^R205G^ Gja3* double mutant (*Gja8^R205G/R205G^ Gja3^−/−^*) lenses from 3-week-old littermates. The *Gja8^R205G/R205G^ Gja3^+/+^* lens section showed disorganized peripheral fibers with vacuoles or enlarged extracellular spaces (labeled with asterisk) near the lens bow region (indicated by an arrowhead), while the double mutant lens section shows organized and elongated periphery fiber cells at the bow region (indicated by an arrowhead). Scale bar, 50 µm.

Previous studies have shown that other *Gja8* mutations display phenotypic variations and the genetic backgrounds of mouse strains influence the severity of cataracts caused by connexin mutations [30], [31], [32]. In order to minimize the influence of strain background and to compare this mutant mouse line with connexin knockout mice in the C57BL/6J stain background, the *Gja8^R205G^* mutant mice were backcrossed into the C57BL/6J strain background for eight generations. We found that heterozygous *Gja8^R205G/+^* mice had normal sized eyeballs and slightly smaller lenses with very mild nuclear cataracts (Figure 2A). Homozygous *Gja8^R205G/R205G^* mice developed microphthalmia with much smaller lenses, dense nuclear cataracts and aberrant vacuole-like structures in the lens periphery (Figure 2A). Homozygous *Gja8^R205G/R205G^* mice displayed much more severe phenotypes than *Gja8^−/−^* mice. These results suggest that the *Gja8^R205G^* mutation still led to semi-dominant lens phenotypes in the C57BL/6J strain background. It is known that Cx50 and Cx46 can form heteromeric and heterotypic channels in the lens [33]. We hypothesized that mutant Cx50-R205G proteins probably act as dominant suppressors through heteromeric or heterotypic interactions to inhibit the functions of wild-type Cx46 connexins thereby causing severe lens phenotypes. We performed a series of experiments to evaluate genetic, biochemical and physiological interactions between mutant Cx50-R205G and wild-type Cx50 or Cx46 *in vivo* and *in vitro*.

### The Cx50-R205G mutant protein perturbs the function of Cx46 in vivo

We first tested whether the severe disruption of peripheral fiber cells in *Gja8^R205G/R205G^* mutant lenses was also dependent on the presence of endogenous *Gja3*. The double mutant *Gja8^R205G/R205G^ Gja3^−/−^* mouse line, lacking endogenous wild-type *Gja3,* was generated from the *Gja8^R205G/R205G^* mutant mouse line crossed with the *Gja8^−/−^ Gja3^−/−^* double knockout (*Gja8^tm1/tm1^ Gja3^ tm1/tm1^*) mice. Both *Gja8^R205G/R205G^ Gja3^+/+^* and *Gja8^R205G/R205G^ Gja3^−/−^* mice developed dense nuclear cataracts. However, the *Gja8^R205G/R205G^ Gja3^−/−^* lens had normal lens periphery, differing from the *Gja8^R205G/R205G^ Gja3^+/+^* lens that had severely disrupted peripheral fiber cell organization with vacuoles (Figure 2A). Histological sections confirmed normal peripheral fiber cells in *Gja8^R205G/R205G^ Gja3^−/−^* lenses, but degenerating fibers with aberrant vacuole-like structures or enlarged extracellular spaces were apparent in the peripheral regions of *Gja8^R205G/R205G^ Gja3^+/+^* lenses (Figure 2B). Thus, the presence of endogenous wild-type *Gja3* was required for peripheral fiber cell degeneration and vacuole formation in *Gja8^R205G/R205G^* lenses. In contrast, the *Gja8^R205G/R205G^ Gja3^−/−^* lens had normal elongated fibers in the periphery, similar to that of *Gja8^−/−^ Gja3^−/−^* double knockout lens reported previously [34].

**Figure 3:**
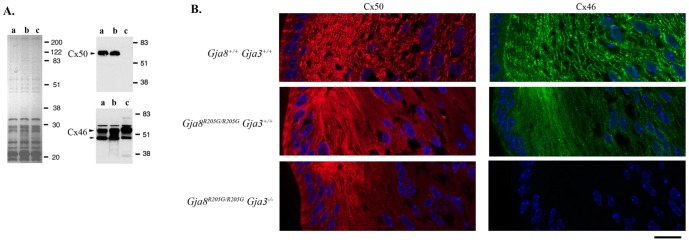
Biochemical and immunological characterization of Cx50 and Cx46 proteins. (**A**) Total lens homogenates, prepared from *Gja8^R205G/R205G^* (a), *Gja8^R205G/−^* (b) and *Gja8^−/−^* (c) littermates at one week of age, were examined by a Coomassie-blue stained gel (left panel) and by western blot using an anti-Cx50 antibody and an anti-Cx46 antibody (right panels). Arrowheads indicate phosphorylated proteins while the arrow indicates non-phosphorylated proteins. Compared to *Gja8^−/−^* lenses, mutant *Gja8^R205G/R205G^* lenses showed decreased phosphorylated Cx46 proteins. (**B**) Immunostaining results of lens frozen sections showed typical punctate signals of Cx50 (red) and Cx46 (green) connexin proteins in wild-type (*Gja8^+/+^Gja3^+/+^*) sample, but only diffuse tiny spots of Cx50 and Cx46 could be seen in *Gja8^R205G^* homozygous mutant (*Gja8^R205G/R205G^ Gja3^+/+^*) sample. Diffuse tiny spots of Cx50 were observed in *Gja8^R205G^ Gja3* double mutant (*Gja8^R205G/R205G^ Gja3^−/−^*) sample. These lens samples were prepared from 1-week-old mice. Scale bar, 20 µm.

Posttranslational modifications, such as phosphorylation, reflect the regulation of connexin proteins *in vivo* [35]. Previous studies reported that the phosphorylated Cx46 protein was not affected in Cx50 knockout (*Gja8^−/−^*) lenses [16], [22] while phosphorylated Cx46 level was decreased in Cx50-G22R (*Gja8^G22G/G22R^*) mutant lenses [36]. Thus, we examined Cx50-R205G mutant proteins and Cx46 wild-type proteins in *Gja8^R205G/R205G^*, *Gja8^R205G/−^* and *Gja8^−/−^* lenses by western blot (Figure 3A). Quantitative densitometric data revealed about a 2-fold reduction in the phosphorylated Cx46 proteins in both *Gja8^R205G/R205G^* and *Gja8^R205G/−^* mutant lens homogenates (n = 3) when compared to *Gja8^−/−^* lenses (arrowhead in Figure 3A). The similar reduction of phosphorylated wild-type Cx46 in both *Gja8^R205G/R205G^* and *Gja8^G22R/G22R^* mutant lenses indicated that Cx50-R205G mutant proteins, like Cx50-G22R mutant proteins, might interact with wild-type Cx46 to affect the regulation of Cx46 proteins. Thus, we further examined the gap junctions in different mutant lenses by immunohistochemical staining.

Gap junction channels consisting of Cx50 and/or Cx46 show typical punctate signals in lens fiber cells by immunostaining. A loss of Cx50 does not affect the typical punctate staining signals of Cx46 in *Gja8^−/−^* lens fiber cells [16], [22], and similarly, a deletion of Cx46 does not affect the typical punctate signals of Cx50 in *Gja3^−/−^* lens fiber cells [21]. Immunohistochemical staining was performed to examine the distribution of Cx50 and Cx46 proteins in lens frozen sections of wild-type (*Gja8^+/+^ Gja3^+/+^), Gja8^R205G/R205G^ Gja3^+/+^* and *Gja8^R205G/R205G^ Gja3^−/−^* mice (Figure 3B). In wild-type lens fiber cells, both Cx50 and Cx46 displayed typical punctate signals (top panels in Figure 3B). However, both Cx50-R205G mutant proteins and Cx46 wild-type proteins showed tiny punctate and diffuse signals in lens fiber cells of the *Gja8^R205G/R205G^ Gja3^+/+^* section, and Cx50-R205G mutant proteins alone also showed tiny punctate and diffuse signals in lens fiber cells of the *Gja8^R205G/R205G^ Gja3^−/−^* section. Thus, regardless of the presence or absence of wild-type Cx46, mutant Cx50-R205G proteins failed to form normal gap junctions in lens fiber cells. Altered distribution and phosphorylation of endogenous wild-type Cx46 proteins support the notion that Cx50-R205G mutant proteins might oligomerize with endogenous wild-type Cx46 to disrupt gap junction formation *in vivo*.

### Cx50-R205G needs the presence of Cx46 to disrupt lens epithelial and fiber cells

**Figure 4:**
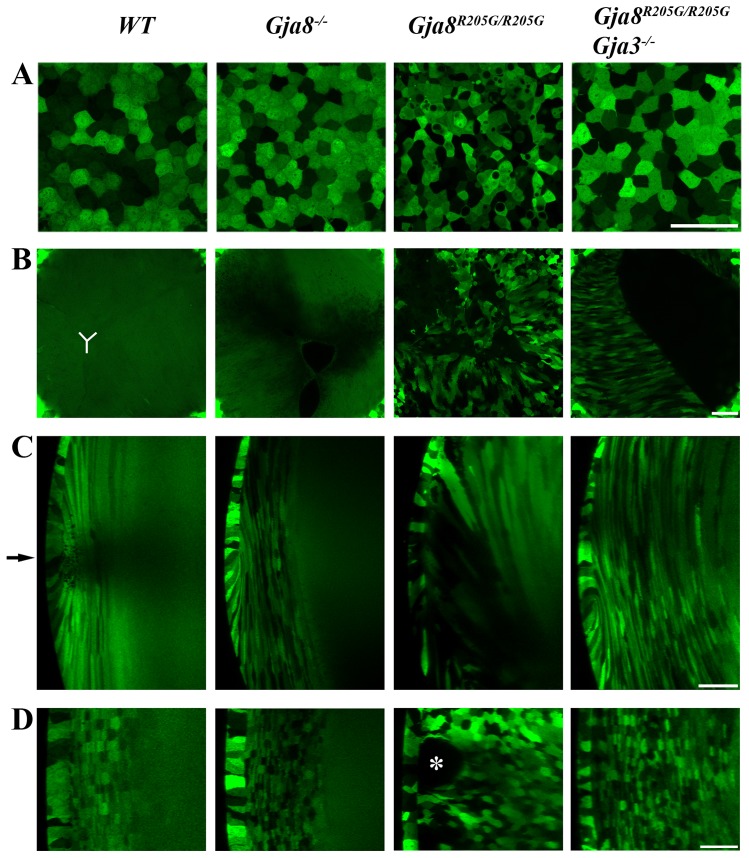
Confocal imaging of GFP-positive (GFP+) live lenses from wild-type (*WT*), *Gja8* knockout (*Gja8^−/−^*), *Gja8^R205G^* homozygous mutant (*Gja8^R205G/R205G^*) and *Gja8^R205G^ Gja3* double mutant (*Gja8^R205G/R205G^ Gja3^−/−^*) mice at the age of 2 **weeks.** (**A**) Confocal images showed the typical mosaic pattern of GFP in lens central epithelial cells in all lenses examined. Aberrant epithelial cells appeared only in the *Gja8^R205G/R205G^* lens. (**B**) Images displayed the anterior Y-suture (delineated by the white lines in the *WT* lens) where the ends of underlying fiber cells come into contact. These elongated fiber cells in the *WT* lens had normal uniform GFP signal. There was a partial delay in Y-suture closure in the *Gja8^−/−^* lens. In the *Gja8^R205G/R205G^* mutant lens, aberrant GFP+ cells were observed in the open suture region where the opposing fiber cells failed to elongate fully to close the anterior Y-suture. The *Gja8^R205G/R205G^ Gja3^−/−^* double mutant lens also had an open suture (black area) due to insufficient fiber cell elongation. (**C**) An anterior (top) to posterior (bottom) sectional view from three-dimensional z-stack reconstructions of GFP+ lenses showing the lens equator (indicated by an arrow) and peripheral fiber cells. Only *Gja8^R205G/R205G^* homozygous lenses displayed disorganized fiber cells. Scale bars, 50 μm. (**D**) A cross sectional view from three-dimensional reconstructions of z-stacks of GFP+ lenses at the lens equator. Lens epithelial cells are on the left. In the *WT* lens, peripheral fiber cells were precisely organized and displayed a mosaic GFP expression pattern while inner fiber cells showed uniform GFP signal. In the *Gja8^−/−^* lens, peripheral fiber cells remained organized. In the *Gja8^R205G/R205G^* homozygous mutant lenses, peripheral fiber cells were completely disorganized with vacuole-like structures or enlarged extracellular spaces (indicated by an asterisk). However, in *Gja8^R205G/R205G^ Gja3^−/−^* double mutant lenses, peripheral fiber cells were mainly organized.

In order to evaluate the potential interactions between Cx50-R205G and Cx46 *in vivo,* we carried out a combined approach including a live lens morphological examination and a genetic test. To determine the cellular alterations underlying cataractogenesis in *Gja8^R205G/R205G^* homozygous mutant mice, we applied a confocal imaging approach using GFP-positive (GFP+) live lenses as reported previously [37]. This approach allows direct morphological observation of changes in lens epithelial cells and fiber cells as well as lens suture formation and the distribution of macromolecules in lens interior fiber cells [38]. The GFP transgene was bred into different genetic mutant mice, and GFP+ live lenses from wild-type, *Gja8^−/−^*, *Gja8^R205G/R205G^* and *Gja8^R205G/R205G^ Gja3^−/−^* mice were collected for imaging analysis (Figure 4).

Normal epithelial cells were observed in either wild-type, *Gja8^−/−^* and *Gja8^R205G/R205G^ Gja3^−/−^* lenses, while aberrant morphological changes and vacuoles appeared in epithelial cells of *Gja8^R205G/R205G^* lenses (Figure 4A). Compared to a typical anterior Y-shape suture in the wild-type lens, the *Gja8^−/−^* lens had a Y-shape suture except the ends of opposing elongating fibers were only partly in contact. However, both *Gja8^R205G/^* ^R205G^ and *Gja8^R205G/R205G^ Gja3^−/−^* lenses had wide open sutures indicating that fiber cells never fully elongated to touch each other under the anterior pole (Figure 4B), which was identical to *Gja8^−/−^ Gja3^−/−^* double knockout lenses reported previously [34], [39]. The identical defects between *Gja8^R205G/R205G^ Gja3^−/−^* and *Gja8^−/−^ Gja3^−/−^* double knockout lenses indicate that Cx50-R205G alone is likely a loss-of-function mutation.

Interestingly, only *Gja8^R205G/^* ^R205G^ lenses (with endogenous wild-type Cx46) displayed substantial aberrant cells in the open anterior suture area. Moreover, GFP+ lens images viewed from the anterior-posterior (AP) axis (Figure 4C) and the cross axis (Figure 4D) revealed severely disrupted peripheral fiber cells with vacuole-like structures or enlarged extracellular spaces in *Gja8^R205G/R205G^* lenses but not in *Gja8^R205G/R205G^ Gja3^−/−^* lenses. This result is consistent with histological data (Figure 2B). Thus, unique pathological changes in epithelial and fiber cells of *Gja8^R205G/R205G^* lenses suggest the genetic interaction between mutant *Gja8* and wild-type *Gja3 in vivo*. Presumably this genetic interaction depends on the direct interactions between Cx50-R205G mutant proteins and wild-type Cx46 proteins in lens cells. Thus, we hypothesized that Cx50-R205G mutant proteins disrupt lens epithelial cells and peripheral fiber cells by a new and adverse gain-of-function interaction with endogenous wild-type Cx46 proteins. We tested this hypothesis by performing electrophysiological studies *in vitro*.

### Cx50-R205G proteins differentially affect the channel functions of wild-type connexins *in vitro*

**Figure 5:**
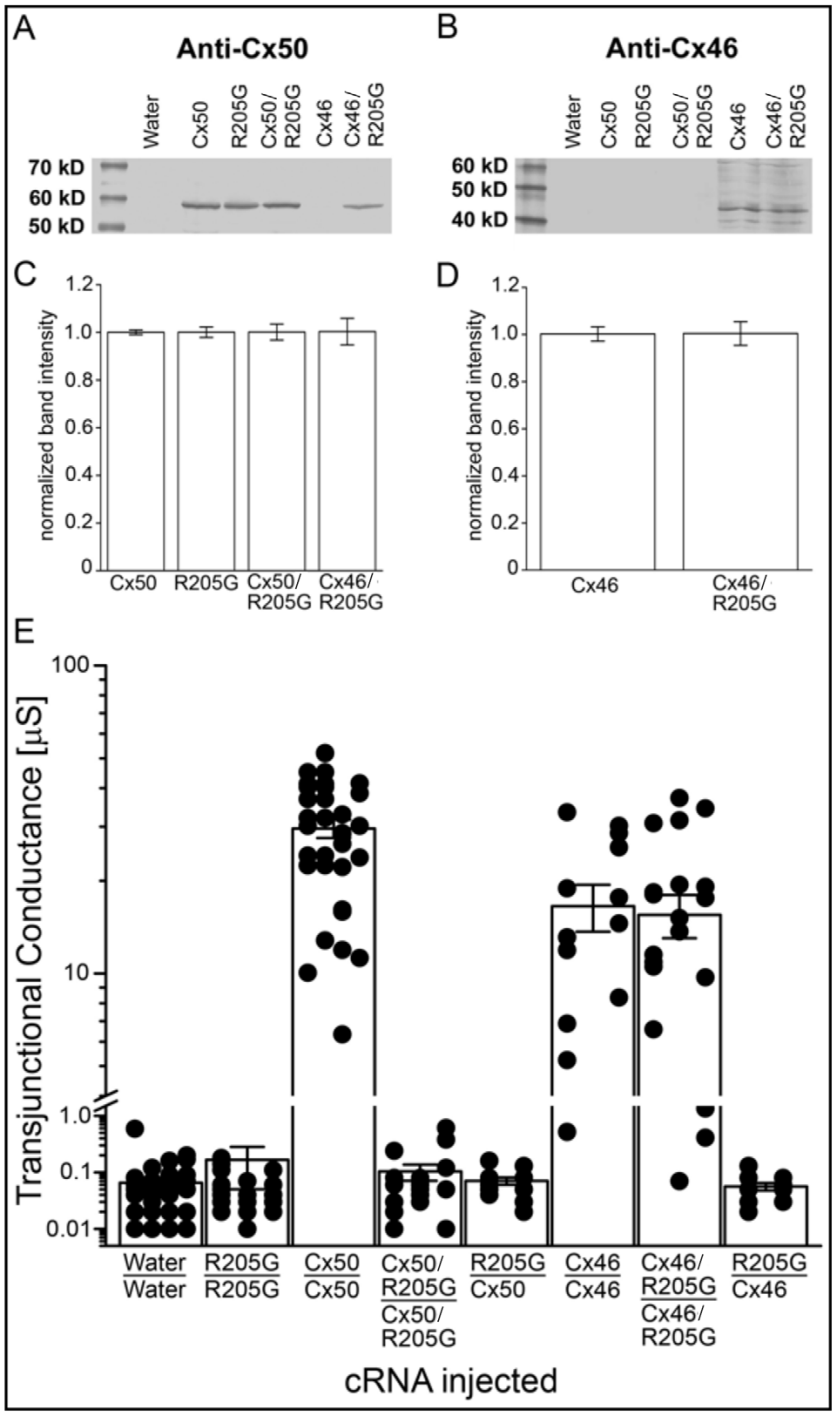
Electrophysiological assays of wild-type Cx50 and Cx46 connexins and mutant Cx50-R205G subunits in paired *Xenopus* oocytes. (**A** and **B**) Western blot analyses of oocytes showed equivalent levels of wild-type and mutant Cx50 when expressed alone or in co-injected cells (A). Western blot also demonstrated similar levels of wild-type Cx46 in both conditions assayed (B). (**C and D**) Band densitometry quantitatively confirmed that mean protein expression was not significantly changed (P>0.05). (**E**) Junctional conductance measurements recorded from *Xenopus* oocyte pairs injected with wild-type *Gja8*, wild-type *Gja3* or mutant *Gja8^R205G^* transcripts alone or in combination. Cell pairs expressing wild-type Cx50 or Cx46 subunits alone formed functional gap junctions with mean conductance values of approximately 30µS and 17µS, respectively. Oocytes co-injected with both wild-type *Gja8* and mutant *Gja8^R205G^* transcripts failed to form functional gap junction channels and exhibited a level of coupling not significantly higher than that of the water-injected control (P>0.05). Conversely, the co-expression of Cx46 and Cx50-R205G subunits did not significantly alter junctional conductance (P>0.05) as these channels displayed a mean G_j_ of 16 µS. Heterotypic channels failed to form functional channels with levels of conductance significantly higher than that of the water-injected controls (P>0.05). Cx50-R205G subunits alone failed to produce functional intercellular channels.

To directly verify the physiological interactions between mutant Cx50-R205G proteins and wild-type Cx50 and Cx46 connexins, we performed electrophysiology assays by using wild-type connexins and mutant Cx50-R205G subunits expressed in paired *Xenopus* oocytes. Both wild-type and mutant genes were subcloned and transcribed *in vitro*. These cRNA transcripts were then microinjected into pre-treated *Xenopus* oocytes that lack endogenous connexin protein subunits, and protein expression of these injected cRNA was examined by western blot analysis. Immunoblot data showed that both wild-type and mutant connexin proteins were produced in oocytes injected with wild-type connexin transcripts alone or co-injected with both wild-type and mutant connexin cRNAs (Figure 5A and 5B). Connexin protein expression was quantitatively analyzed by band densitometry (Figure 5C and 5D). These data revealed that wild-type and mutant protein levels were not significantly different in oocytes injected with wild-type or mutant connexin cRNAs alone or in cells co-injected with both wild-type and mutant connexins (P>0.05). Therefore, any alteration in channel function was not a result of a change in connexin expression or an increase in protein degradation for any of the conditions assayed.

We then measured junctional conductance of paired oocytes expressing connexin proteins using a dual whole-cell voltage clamp (Figure 5E). Data showed that homotypic wild-type Cx50 and Cx46 channels displayed mean conductance values of 29 µS and 16.5 µS, respectively. In contrast, pairs containing Cx50-R205G subunits alone failed to electrically couple cells, displaying conductance that was not significantly higher than water-injected negative controls (P>0.05). Similarly, heterotypic cell pairs, in which one cell expressing wild-type Cx50 or Cx46 subunits and the opposing cell expressing Cx50-R205G subunits, were also not electrically coupled. The co-expression of wild-type and mutant connexins allowed us to examine the heteromeric interactions between mutant and wild-type subunits. A quantitative comparison of the mean conductance between homotypic Cx46 channels and channels made from a mixture of Cx46 and Cx50-R205G subunits revealed no significant reduction in electrical conductance (P>0.05). In contrast, the mean conductance of channels made from a mixture of wild-type Cx50 and mutant Cx50-R205G revealed no coupling, similar to water-injected negative controls. These data indicate that the mutant Cx50-R205G protein acts as a dominant negative repressor for channel conductance of wild-type Cx50, but not Cx46, in paired oocytes *in vitro*.

**Figure 6:**
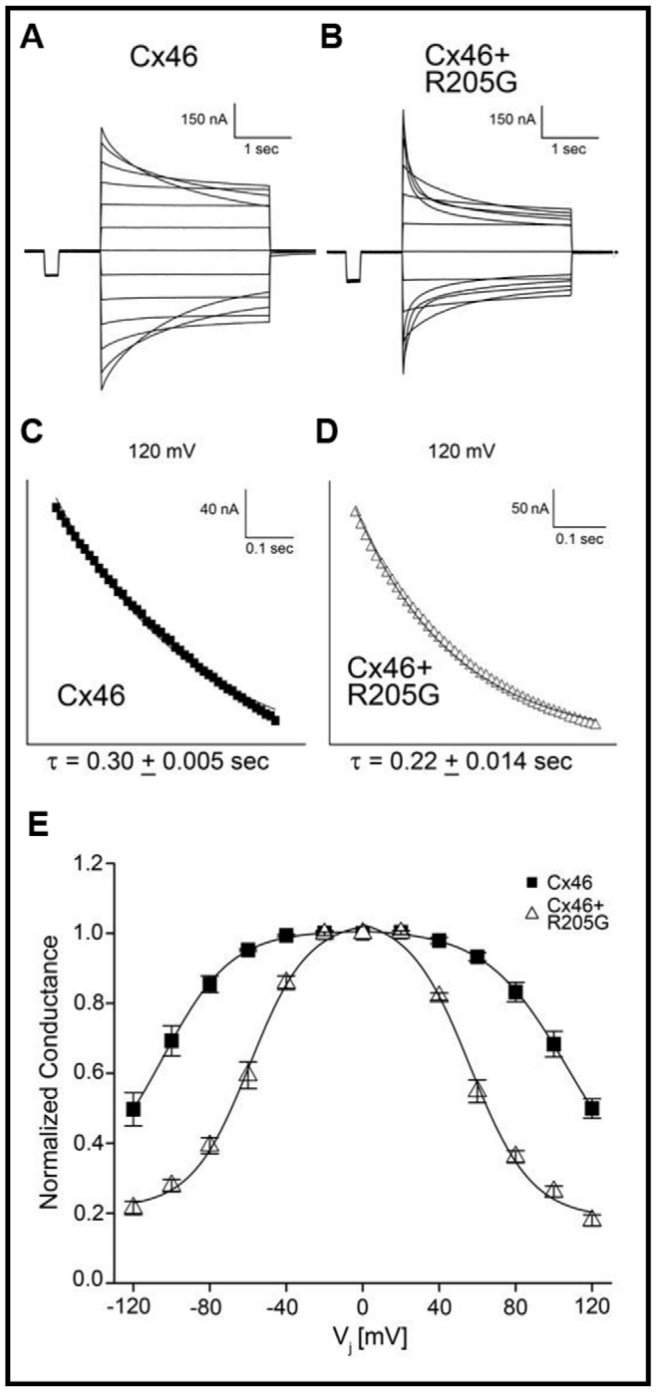
Voltage-gating properties of Cx46 and mixed Cx46/Cx50-R205G channels. The decay in junctional current (I_j_) induced by transjunctional voltage (V_j_) was plotted as a function of time for gap junctions comprised of Cx46 (**A**) and mixed Cx46/Cx50-R205G (**B**). Voltage was stepped to ±120 mV in 20 mV increments. At all voltage applications >±20 mV, mixed channels showed a more rapid current decay toward steady state. Analysis of channel closure kinetics was based on representative traces displaying the initial 250 ms of current decay recorded after application of a +120 mV transjunctional voltage, for gap junctions comprised of homomeric wild-type Cx46 (n = 5) (**C**) and heteromeric channels containing both Cx46 and Cx50-R205G (n = 5) (**D**). Current traces were fit to monoexponential decay to determine the mean time constant, τ. Heteromeric Cx46 and Cx50-R205G channels closed ∼25% faster than homomeric Cx46 channels, displaying a significant increase in mean channel closure time (p<0.05). (**E**) Comparison of equilibrium conductance. Steady state conductance was measured when current decay reached equilibrium, normalized to the values at ±20 mV and plotted as a function of V_j_. The steady state reduction in conductance for heteromeric Cx46 and Cx50-R205G channels (n = 8) was greater than the reduction for homomeric wild-type Cx46 channels (n = 5).

Since genetic, immunological and biochemical data suggested the interaction between Cx50-R205G and endogenous wild-type Cx46, we further tested whether Cx50-R205G mutant proteins might be capable of forming heteromeric channels with wild-type Cx46 in *Xenopus* oocytes and altering the channel gating properties. The voltage gating properties of Cx46 channels with or without Cx50-R205G in *Xenopus* oocytes were analyzed (Figure 6). The application of a series of transjunctional voltages (0 mV to ±120 mV) caused a slow decay of junctional current toward steady state at all potentials analyzed in homomeric Cx46 channels (Figure 6A). Conversely, oocytes co-injected with Cx46 and Cx50-R205G transcripts exhibited a visible change in the rate of Ij decay when compared to homomeric Cx46 channels (Figure 6B). This qualitative change was supported by a quantitative examination of channel gating kinetics. The initial 300 milliseconds of Ij decay was fit by a monoexponential decay function and the time constant τ was determined (Figure 6C). The data revealed that wild-type Cx46 and Cx50-R205G heteromeric channels closed significantly faster than homomeric Cx46 channels (P<0.05), as the mean τ value for co-injected pairs was ∼215 milliseconds, a value over 100 milliseconds faster than that of the wild-type Cx46 channels (Figure 6D). The steady state junctional conductance of Cx46 channels and Cx46/Cx50-R205G mixed channels was measured during the application of voltage pulses ranging from ±20 to ±120 mV, recordings were then normalized to the values obtained at ±20 mV, and plotted against V_j_. The data showed that the reductions in equilibrium conductance for Cx46/Cx50-R205G mixed channels were greater at all tested voltages than the reductions for Cx46 channels (Figure 6E). In addition, Cx46/Cx50-R205G heteromeric channels exhibited about 50% decrease in the values of V0 compared to Cx46 homomeric channels (Table 1). The shifted gating properties of Cx46/Cx50-R205G heteromeric channels suggest that Cx50-R205G may also alter the function of Cx46.

**Table 1:** Boltzmann parameters for homomeric Cx46 and heteromeric Cx46/Cx50-R205G channels.

Connexon	V_j_	V_0_	G_jmin_	A
Cx46	+	107	0.21	0.04
Cx46	−	−105	0.28	0.04
R205G+Cx46	+	56	0.19	0.05
R205G+Cx46	−	−59	0.21	0.06

*G_jmin_* represents the minimum conductance value, *V_0_* indicates the voltage measured midway through the G_j_ decline, and *A* denotes the cooperativity constant, reflecting the number of charges moving through the transjunctional field. Signs + and – for V_j_ indicate transjunctional membrane potential polarity.

## Discussion

This work reveals genetic, molecular, cellular, and physiological evidence about how the *Gja8^R205G^* missense point mutation causes lens phenotypes in *Nm2249* mutant mice [28]. The R205G mutation alters at least three functional properties of Cx50 proteins. First, Cx50-R205G mutant proteins alone are unable to form functional channels in the lens and in the paired oocytes. Second, Cx50-R205G mutant proteins act as dominant suppressors to block the conductance of wild-type Cx50 channels and to affect the gating of heteromeric channels with wild-type Cx46. Third, the heteromeric interaction between mutant Cx50-R205G proteins and endogenous wild-type Cx46 shifts the channel gating properties that may lead to the disruption of lens peripheral fibers and epithelial cells. Moreover, the R205 residue in the mouse Cx50 protein is orthologous to the R198 residue in the human Cx50 protein. This work will be useful for understanding dominant congenital cataract-microcornea syndrome (CCMC) in human patients caused by the *GJA8^R198W^* mutation [29].

The R205 is a conserved residue in the connexin superfamily located in the extracellular loop 2 (EL2) of the mouse Cx50 protein. SIFT, Polyphen2, and Condel all predict that mouse R205G (or human R198G) change will cause major problems (data not shown). Channel formation relies on the appropriate docking of these extracellular loops of two apposed connxons (hemichannels). EL2 is indicated to play a role for the docking selectivity of heterotypic interactions between different connexin subunits [40]. To date, *Gja8^R205G^* is the first mouse point mutation identified in EL2. It is likely the R205G mutation perturbs selective docking of EL2 in gap junction formation, and that may explain how mutant Cx50-R205G proteins act as a dominant negative inhibitor that specifically blocks channel conductance of wild-type Cx50, but only affects channel gating of Cx46 in paired *Xenopus* oocytes. In contrast to normal and clear lenses in *Gja8* ^+/−^ mice, Cx50-R205G mediated inhibition of endogenous wild-type Cx50 function may be the cause of smaller lenses of heterozygous *Gja8^R205G/+^* mutant mice, similar to *Gja8* ^−/−^ homozygous knockout lenses [16], [22]. In contrast, both the *Gja8^G22R^* mutation in the N-terminus and the *Gja8^S50P^* mutation in extracellular loop 1 inhibit the channel conductance and gating of wild-type Cx46 in paired *Xenopus* oocytes [24], [25]. This difference may contribute to the phenotypic differences among these different *Gja8* mutant mice.

Immunostaining results showed that all three Cx50 mutants (*Gja8^R205G^*, *Gja8^S50P^* and *Gja8^G22R^*) affect gap junction formation consisting of wild-type Cx46 by altering typical punctate signals into diffuse tiny spots in the lens fibers. Cx50 and Cx46 can form heteromeric hemichannels in the lens [33]. It is known that oligomerization of connexin subunits to form connexon hemichannel occurs in ER and/or Golgi [41]. Decreased level of phosphorylated Cx46 proteins and a change of Cx46 staining pattern in mutant lens fiber cells suggest interactions between mutant Cx50 protein subunits and wild-type Cx46 protein subunits. It is possible that the disruption of the gap junction formation by endogenous Cx46 is partly a result of altered protein trafficking or channel assembly of heteromeric connexons consisting of Cx50 mutant subunits and wild-type Cx46 subunits [36], [39].

In summary, this work defines unique and common functional changes that are associated with the *Gja8^R205G^* mutation. The mutant Cx50-R205G protein acts as a loss-of-function mutation in the absence of endogenous wild-type connexins, displays dominant inhibition toward wild-type Cx50 and alters wild-type Cx46 in different manners. These results provide some mechanistic information to explain many *Gja8* mutations that have been linked to dominant cataracts in humans and mice [4]. Future studies will be needed to further delineate different molecular mechanisms for cataracts caused by different Cx50 point mutations.

## Materials and Methods

### Gene mapping, sequencing and PCR genotyping of different mutant mice

Animals were cared for in accordance with the ARVO statement for the Use of Animals in Ophthalmic and Vision Research, and all studies were conducted in accordance with a protocol approved by the Animal Care and Use Committee (ACUC) at University of California, Berkeley.

The CWXS/Agl, a recombinant inbred strain between CWD/LeAgl and SJL/J, imported from Dr. Angel at MD Anderson Cancer Center at University of Texas in 1995, is a spontaneous mutant strain with microphthalmia and cataract. When CWXS/Agl arrived at The Jackson Laboratory, it was assigned JR #2801 (JR2801). After its cataract phenotype was confirmed, this mouse mutant line was renamed as new mutant number 2249 (*Nm2249*). To map the chromosomal location of the cataract mutant gene, *Nm2249* mutant mice in the CWXS/Ag1 strain background were mated with CAST/Ei mice to generate 105 backcrossed mice. Tail DNA was isolated for linkage analysis, and these backcrossed mice were phenotyped and genotyped by a genome-wide screen used previously [36]. Loci showing significant skewing of the alleles were identified and additional markers around them were selected for fine mapping. Two pairs of PCR primers (Gja8-F and Gja8-R, Gja8-2F and Gja8-2R) based on the mouse *Gja8* gene sequence in GenBank (accession number M91243) covering the whole coding region were designed for sequencing. Primer sequence: Gja8-F, 5′-ggcacttgatagaagctgttgg-3′ (125–146), Gja8-R, 5′-ggtggaccaagggacacataag-3′ (1690–1669), Gja8-2F, 5′-ggccattacttcctgtatgg-3′ (789–808) and Gja8-2R, 5′-ttctcagcaatctccccagtg-3′ (1036–1015).

Standard PCR genotyping methods were used to genotype the knockout, mutant or wild-type alleles of *Gja8* and *Gja3*. A 300bp fragment was expected for wild-type *Gja3* using the primer pair 5′-cccaggctctacctcaggtt-3′ (sense) and 5′-ctttgccgatgactgtagag-3′ (antisense); a 500bp fragment for knockout *Gja3* was produced by the primer pair 5′-cccaggctctacctcaggtt-3′ (sense) and 5′-cagggttttcccagtcacgac-3′ (antisense); a 320bp fragment for either the *Gja8^R205G^* or wild-type allele of *Gja8* was amplified with the primer pair 5′-ggatcctttcaaacaac-3′ (sense) and 5′-gccgatgacagtggagtgctc-3′ (antisense); a 450bp fragment for the knockout allele of *Gja8* was detected using the primer pair 5′-ggatcctttcaaacaac-3′ (sense) and 5′-cagggttttcccagtcacgac-3′ (antisense).

Mutant *Gja8^R205G^* cDNA was generated from total mRNAs of homozygous mutant lenses by RT-PCR using a pair of primers, sense 5′-cgggatcctagtgagcaatgggcgac-3′ and anti-sense 5′-ggaattcgtcatatggtgagatcatc-3′. DNA sequencing data confirmed that mutant cDNA sequence was identical to the coding region of its genomic DNA. Mutant cDNA was subcloned into pCR-bluntII-TOPO vector (Invitrogen, Carlsbad, CA) and sequenced to confirm the arginine to glycine substitution at codon 205.

### Histological, immunohistochemical and biochemical analyses

Standard histology methods were used for analyzing mutant and normal lenses [39]. Immunohistochemical study was performed by using frozen lens sections with antibody staining, following the procedure described previously [21]. A rabbit polyclonal antibody against the intracellular loop of Cx46 connexin [42] and a rabbit polyclonal antibody against the C-terminal region of Cx50 connexin were used [36]. Fluorescent images were collected by a Zeiss LSM 700 confocal microscope. Enucleated fresh lenses were weighed and homogenized as total protein samples. An aliquot of 20 μg of lens total proteins from each sample was loaded for western blot analysis according to a procedure described previously [21].

### Imaging of GFP-positive live lenses

GFP-positive (GFP+) knockout mice were generated by intercrossing *Gja8^R205G/R205G^* mice with GFP transgenic *Gja8^−/−^* mice in the C57BL/6J strain background [38]. A UV lamp was used to screen GFP+ mice. Mutant mice with one copy of the GFP transgene were used for imaging live lenses using a procedure reported previously [38]. A Zeiss LSM700 confocal microscope with ZEN 2009 software was used to collect and process images from fresh intact GFP+ lenses. Z-stacks were collected at 1 µm steps through anterior and the equator of the lens.

### Electrophysiological assay in paired *Xenopus* oocytes

Mutant *Gja8^R205G^* cDNA was subcloned into the pCS2+ and pIRES2-EGFP expression vectors (Clontech, Palo Alto, CA) using the EcoR1 restriction sites for expression in *Xenopus laevis,* sequencing and immunofluorescent staining. Wild-type mouse *Gja3* was inserted into the pCS2+ expression vector using the *XhoI* and *SpeI* restriction sites. The wild-type *Gja8*, *Gja3* and mutant *Gja8^R205G^* plasmids were linearized at the *NotI* restriction site of pCS2+, and transcribed using the SP6 mMessage mMachine (Ambion). Adult *Xenopus* females were anesthetized, the ovaries were removed and Stage V-VI oocytes were collected after the ovarian lobes were de-folliculated in a solution containing 50 mg/ml collagenase B and 50 mg/ml hyaluronidase in modified Barths medium (MB) without Ca^2+^. Oocytes express only Cx38, but not Cx50 or Cx46.

To eliminate the possible contribution of endogenous intercellular channels, oocytes were first injected with 10 ng of antisense *Xenopus* Cx38 oligonucleotide (5′ CTGACTGCTCGTCTGTCCACACAG-3′) to eliminate coupling caused by endogenous intercellular channels and cultured overnight in MB medium containing 2 mM CaCl_2_. Oligonucleotide injected oocytes were then injected with either wild-type or mutant *Gja8* cRNA transcripts (5 ng/cell) alone, in combination or with water as a negative control. The vitelline membranes were then removed in a hypertonic solution (200 mM aspartic acid, 10 mM HEPES, 1 mM MgCl_2_, 10 mM EGTA, and 20 mM KCl, pH 7.4), and the oocytes were manually paired with the vegetal poles apposed in either normal MB media or MB with elevated Ca^2+^ (2 mM CaCl_2_). Oocyte pairs were prepared and measured using the dual whole-cell voltage clamp technique [43]. Functional conductance, voltage-gating properties and transjunctional potentials (Vj) were measured by a procedure reported previously [24], [25]. Macroscopic recordings of hemichannel currents were obtained from single Xenopus oocytes using a GeneClamp 500 amplifier controlled by a Digidata 1320 interface (Axon Instruments, Foster City, CA). Electrophysiological recording of hemichannel currents was carried out as reported previously [25].

### Preparation of oocyte samples for western blot analysis

Oocytes were collected in 1 ml of buffer containing 5 mM Tris pH 8.0, 5 mM EDTA and protease inhibitors [44] and lysed using a series of mechanical passages through needles of diminishing caliber (20, 22 and 26 gauges). Extracts were centrifuged at 1000 *g* at 4°C for 5 minutes. The supernatant was then centrifuged at 100,000 *g* at 4°C for 30 minutes. Membrane pellets were resuspended in SDS sample buffer (2 µl per oocyte), and samples were separated on 10% SDS gels and transferred to nitrocellulose membranes. Blots were blocked with 5% BSA in 1X PBS with 0.02% NaN_3_ for 1 hour and probed with a polyclonal Cx50 antibody or a polyclonal Cx46 antibody followed by incubation with alkaline-phosphatase conjugated anti-rabbit secondary antibody. Band intensities were quantified using Kodak 1D Image Analysis software (Eastman Kodak, Rochester, N.Y., USA). Values from four independent experiments were normalized to the mean value of band intensity of the wild-type sample.
